# Enhancing Sensory Experiences for Infants Born Preterm: A Quality Improvement Project

**DOI:** 10.1016/j.arrct.2024.100377

**Published:** 2024-10-26

**Authors:** Kate N. de Castro Mehrkens, Elena V. Donoso Brown

**Affiliations:** a Rehabilitation Department, Atrium Health Floyd, Rome, GA; b Department of Occupational Therapy, Duquesne University, Pittsburgh, PA

**Keywords:** Child development, Neonatal intensive care unit, Neonate, Occupational therapy, Rehabilitation

## Abstract

**Objective:**

To enhance neonatal sensory experiences in infants born preterm: a quality improvement project.

**Design:**

This was a time-interruption quality improvement project. The time-interruption lasted 4 weeks with an 8-week data collection period before and after. Baseline data were collected on current program use for 8 weeks prior to the time interruption. The neonatal intensive care unit (NICU) occupational therapist provided staff education, training, and modeling of the Supporting and Enhancing NICU Sensory Experiences (SENSE) program during the time interruption. Data were collected for 8 weeks after the time interruption to track improvements.

**Participants:**

Twenty infants and their families participated, as well as NICU nurses who provided positive sensory experiences and completed surveys.

**Main Outcome Measures:**

Data related to delivery of, education on, and adherence to SENSE were collected before, during, and after the time interruption. Staff surveys were completed pre- and posteducation, training, and modeling to gather perspectives on program usage.

**Results:**

On average, parents delivered 43.88 minutes of positive sensory input per day before and increased to 92.7 minutes per day after the time interruption. Positive tactile recommendations were met, on average, 19% of the time before and 47% of the time after the time interruption. After education, training and modeling, nursing staff unanimously supported the program according to surveys.

**Conclusions:**

Although a higher percentage of infants received the recommended dosage of positive tactile input after intervention, opportunities still exist to improve and expand implementation.

## Highlights

• SENSE can be incorporated to increase positive sensory experiences in the NICU
• Strategies to educate, train, and model SENSE to nursing staff increased its use
• SENSE supported unanimously by the parents/guardians and the nursing staff surveyed
• The is still more expansion of the program needed to improve adherence

In the United States, 9.07% of infants go to the neonatal intensive care unit (NICU) as a result of preterm birth, complex delivery, or medical complications.^1^^,^^2^ The interdisciplinary NICU team supports healing, growth, and development.^3^ Although medical care is critical, research demonstrates the NICU environment can hinder preterm infants’ neuro-sensorimotor development. The lack of positive sensory experiences, coupled with noxious stimuli, affect the developing nervous system and can increase the risk of long-term delays.^4^, ^5^, ^6^, ^7^, ^8^, ^9^, ^10^

Positive sensory experiences in the NICU are evidence-based interventions to mitigate negative outcomes. Holding skin to skin, providing positive physical and tactile input, and exposing infants to language releases oxytocin, thereby reducing cortisol levels.^11^ Preterm infants have improved motor development and lower stress when parents are consistently present and hold their child.^12^ Additionally, preterm infants who are held more frequently have better reflex development.^13^

Some existing programs to provide positive sensory experiences and support infant development include hospital-specific developmental care programs,^14^ the Newborn Individual Developmental Care and Assessment Program,^15^ and the Supporting and Enhancing NICU Sensory Experiences (SENSE) program. This project was modeled after the SENSE program. SENSE was created to provide dosed multimodal sensory interventions to support neurodevelopment in medically fragile infants through (1) caregiver education and (2) recommendations for appropriate positive sensory experiences for infants born preterm.^16^^,^^17^ The recommended sensory dosages are based on current evidence and are frequently reviewed.^18^ Education topics include infant development, parenting, reading infant cues, and delivering positive sensory interventions.

SENSE has resulted in improvements in infant development,^19^ maternal confidence/parent comfort,^19^ parent satisfaction,^20^ and increased sensory experiences.^21^^,^^22^ Despite these benefits, the literature outlines various challenges involved in implementing SENSE including time commitment, staff involvement, and difficulty monitoring and achieving sensory dosages.^20^^,^^23^

The goal of this quality improvement (QI) project was to determine if training, educating, and modeling the use of SENSE to NICU nursing staff would improve program implementation in a rural area NICU.

## Methods

### Context

This QI project took place in a 23-bed, level III NICU. One full-time occupational therapist, the first author, is present Monday through Friday from 7:30 am to 3:30 pm. The occupational therapy caseload ranges from 4-20 infants who receive services 3-5 times per week.

### Initial use of SENSE

In spring 2022, the NICU manager, director of rehabilitation, and neonatologists approved the program. Age-appropriate sensory recommendations and educational quick response codes were laminated (for cleaning/reuse), hung at infant bedsides, and updated weekly. When parents/guardians were present, the occupational therapist provided SENSE education. No logbooks or printed educational packets were used (unless a parent/guardian did not have a smartphone, in which case the packet was printed) because past studies have noted a lack of accuracy as well as negative comments from parents.^20^^,^^21^ Table 1 shows the comparison of the original program materials and materials used in this NICU. In the first 6 months, challenges with inconsistent use of SENSE arose, which led to this project. Data collection began after institutional review board approval and does not include these first 6 months.

**Table 1:** Components of SENSE program versus components used in this project.

Variable	SENSE Program	Components of SENSE Used in This Project
Parent/guardian education	Caregiver education printed packets, QR codes, and hanging bedside materials	QR codes and hanging bedside materials*
Sensory experiences	Adhere to SENSE dosage recommendations based on infant's medical status	Adhere to SENSE dosage recommendations based on infant's medical status
Tracking SENSE	May or may not use logbooks	Observation and medical documentation

Abbreviations: QR code, quick response code; SENSE, Supporting and Enhancing Neonatal Intensive Care Unit Sensory Experiences.

⁎ Printed packets were made available if the parent/caregiver was unable to scan a QR code.

### Project design

**Fig 1:**
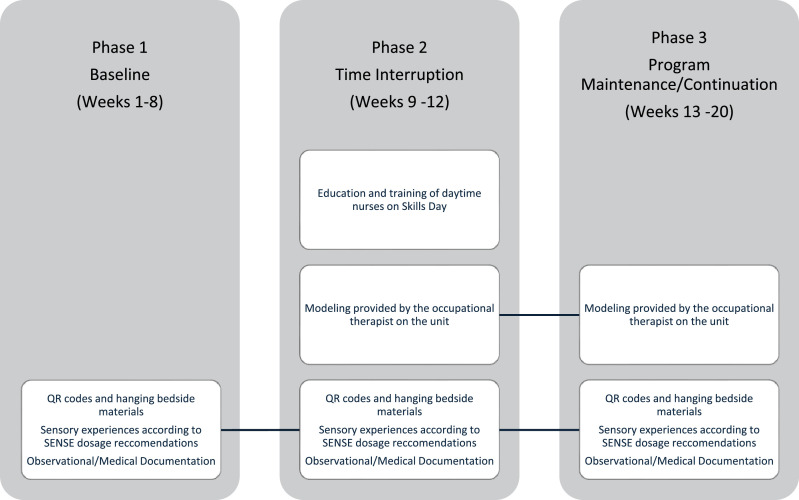
Project timeline. QR code, quick response code.

This project used a time-interruption design, which allowed for data collection before and after interventions were implemented to improve SENSE delivery. The time-interruption design is supported in the literature for community-based studies that have multiple measures recorded across time.^24^ The time interruption lasted 4 weeks (phase 2) with 2 8-week data collection periods before and after (phases 1 and 3). Figure 1 shows a visual of the project design.

### Participants

Twenty infants and their families participated. The only criteria to participate was that the infant received occupational therapy services. Participants had the following diagnoses: preterm birth, poor feeding, neonatal abstinence syndrome/drug exposure during intrauterine life, and/or neurologic diagnoses. Only infants who had a parent(s)/legal guardian(s) present during the occupational therapist's weekday hours could participate because this is when informed consent was obtained. Daytime nurses also participated; however, the institutional review board stated that informed consent was unnecessary for nurses because this was a QI project and data remained anonymous.

### Measures

Several implementation variables were captured to understand the effect of this project. First, the recruitment and retention rates were tracked. Other main measures included (1) delivery of; (2) education on; and (3) adherence to SENSE. Regarding delivery and adherence, only tactile input was measured because this was the most quantifiable measure. Table 2 shows more information on the variables.

**Table 2:** List of variables for each section of data collection (delivery, education, and adherence).

Delivery of SENSE	Education on SENSE	Adherence to SENSE
Number of times parent/guardian visits per day	Number of times OT educates parent/guardians per week	Number of participants who receive the full dosage recommendation of tactile input
Percentage of infants who had a caregiver visit each day (%)	Before staff education survey*	
Length of positive sensory input delivered by parent/guardian each day (min)	After staff education survey*	
Length of positive sensory input delivered by OT every day (min)	Number of times SENSE is modeled by OT to staff†	
Percentage of bedspaces with accurate SENSE materials hanging (%)		
Number of times nurse delivered positive sensory input†		

Abbreviations: OT, occupational therapist; QR code, quick response code; SENSE, Supporting and Enhancing Neonatal Intensive Care Unit Sensory Experiences.

⁎ Phase 2 only.

† Phases 2 and 3.

During phase 2, 2 nonstandardized surveys were used to evaluate daytime nurses’ perspectives. The pre-education survey included questions related to awareness of SENSE and insights regarding appropriate sensory experiences. The posteducation survey related to perspectives on SENSE and willingness to use the program. Because data collection involved observation, only daytime nurses’ use of the program during the occupational therapist's hours were included. See Appendix 1 for the surveys.

### Data collection and intervention

Data were collected from the day the infant was recruited until they were discharged or the project ended. Data for variables related to delivery/adherence to SENSE were collected via observation by the full-time NICU occupational therapist as well as documentation from nurses. The unit has an open layout allowing for observation to occur easily. Swaddled or skin to skin holding (positive tactile input) that occurred outside of the occupational therapist's hours were documented in medical charts by nurses as part of regular documentation. These data were added to the sensory input minutes for each day. The occupational therapist also recorded time she spent delivering positive sensory experiences to the participating infants. Phase 1 included baseline SENSE data collection, phase 2 consisted of data collection during the time interruption, and phase 3 involved data collection related to continuation and maintenance of the program.

#### Phase 1: Baseline SENSE data gathering (weeks 1-8)

The first phase aimed to gather baseline data on current SENSE use. Parents/guardians received education when present and were welcome to provide sensory experiences to their infant(s) when medically stable. Nurses continued to provide typical care to the infants, which included nursing-related caregiving tasks (ie, holding an infant for feeding is not included in positive sensory input delivered by staff). These activities were considered the standard of care.

Data related to parent/guardian education on and delivery of SENSE were collected during this phase. Data for sensory experiences delivered by the occupational therapist were also recorded. No data were collected on nurses’ use SENSE during this phase because they had not yet been formally educated or trained. Finally, the number of infants with accurate sensory recommendations hanging at their bedside was tracked. Table 2 lists the variables used for delivery of, education on, and adherence to SENSE.

#### Phase 2: Time interruption (weeks 9-12)

Daytime nurses were asked to complete the preintervention surveys regarding their perspectives on appropriate dosages of sensory experiences at the start of this phase. The occupational therapist then used the education, training, and modeling interventions supported by the Behavior Change Wheel.^25^ Although other interventions from the Behavior Change Wheel were considered, these interventions were selected because they were the most feasible and relevant for this program.

Education and training interventions took place on NICU Skills Days, which was an annual education requirement for all NICU nurses. At the developmental care station, the occupational therapist provided verbal education and hands-on training about SENSE via verbal explanation, printed SENSE materials, and demonstration. Nurses were required to demonstrate appropriate positioning and handling of a practice doll and verbalized understanding of SENSE. After education, the occupational therapist offered posteducation surveys to daytime nurses.

Modeling took place in the NICU when the occupational therapist demonstrated appropriate use of SENSE to nurses. Daytime nurses also likely heard the occupational therapist delivering education to parents/guardians, which would re-emphasize the education and training provided during Skills Days. Data collection included details on nurses’ use of SENSE along with all variables previously collected in phase 1 (see table 2).

#### Phase 3: Maintenance (weeks 13-20)

During the final phase, the occupational therapist continued modeling SENSE and educating parents/guardians. Data collection continued across all variables outlined in table 2 to monitor changes in program usage after the interventions.

### Data analysis

Deidentified data from each phase and across phases were collected and analyzed in Microsoft Excel.

#### Participant demographics and participation

Demographic data were analyzed with descriptive statistics using frequencies and means. These variables included (1) postmenstrual age on day of recruitment; (2) sex; (3) ethnicity/race; and (4) pregnancies involving multiples. The number of phases in which each infant participated was recorded. Recruitment and retention rates were also recorded as percentages.

#### Delivery of SENSE

Data analysis regarding delivery of SENSE included averages obtained for each week and then across all weeks during each phase. In relation to parent/guardian delivery of SENSE, the following results were calculated: (1) average number of times a parent/guardian visited each day; (2) percentage of participants who had a parent/guardian present each day; and (3) average length (minutes) of positive sensory input (tactile) from parent/guardian.

Regarding delivery of SENSE by staff (recorded during phases 2 and 3), the number of times that nurses delivered positive sensory input (tactile) outside of typical care was summed. Finally, the percentage of bedsides with accurate SENSE sensory recommendations hanging were averaged across all weeks in each phase.

#### Education on SENSE

The average number of times the occupational therapist educated the parent/guardian each week and then across all weeks within each phase was calculated. The number of times the occupational therapist modeled SENSE to staff during phases 2 and 3 were totaled within each phase. Data for the staff surveys were analyzed as percentages for each item.

#### Adherence to SENSE

Percentages related to program adherence were calculated by week and then within each phase. Program adherence was monitored by calculating the percentage of infants who received the full recommended amount of positive tactile input. This included data from parents, daytime nurses, and the occupational therapist delivering positive tactile input.

### Ethical considerations

This project was deemed beneficial because positive sensory experiences for preterm infants are well supported in the literature. All parents/guardians signed an informed consent document prior to participation. All data are deidentified. Because this is a QI project and SENSE is supported in the literature,^19^^,^^20^^,^^22^ infants who did not participate still received age-appropriate positive sensory interventions.

## Results

### Infant demographics and participation

All families who were asked agreed to participate (100% recruitment rate). The number of participants per week ranged from 2-6 infants. The average age of the infants on the day they were recruited to participate was 32.85 weeks (range, 26-37wk). Forty percent of the participants were twins, 55% were men, and 70% were White. Because of lengthy infant hospitalizations, 35% (n=7) participated in ≥2 phases, and 10% (n=2) participated in all 3 phases. All families participated until they were discharged (100% retention rate). Table 3 lists the demographic details of the infants.

**Table 3:** Participant demographic data.

Participant Number	Age on Day of Recruitment (wk)	Sex at Birth	Ethnicity/Race	Multiple Pregnancy	Participation in Phase 1	Participation in Phase 2	Participation in Phase 3
1	37	F	White		*		
2	33	F	Black		*		
3	30	F	White		*		
4	32	F	White		*		
5	33	F	White		*		
6	35	M	White		*		
7	26	M	White		*	*	*
8	34	M	White	Twin	*	*	
9	34	M	White	Twin	*	*	
10	31	M	Black		*	*	*
11	34	F	White		*	*	
12	32	M	White	Twin		*	*
13	32	F	White	Twin		*	*
14	35	F	White				*
15	34	M	Hispanic	Twin			*
16	34	M	Hispanic	Twin			*
17	35	M	Black				*
18	33	M	White	Twin			*
19	33	M	White	Twin			*
20	30	F	Mixed				*

### Delivery of SENSE

The environment had appropriate sensory recommendations hanging at the bedside ≥95% of the time. Daytime nurses were observed providing positive tactile input 6 times during the second phase. Positive tactile input from daytime nurses was observed 21 times in the third phase.

Parent/guardian presence was highest during the second phase, with 82% of infants having a parent/guardian present at least once per weekday each week. Parents/guardians in the second phase also had an average of 1.22 visits per day compared with an average of 0.64 during phase one 1 and 1.05 during phase 3. Positive sensory (tactile) input from parent(s)/guardian(s) increased from an average of 43.88 minutes per day (phase 1) to 98.68 minutes per day (phase 2) and then dropped to an average of 92.7 minutes per day (phase 3). See table 4 for averages and the supplement for individual data.

**Table 4:** Average data across variables within the 3 data collection categories: delivery, education, and adherence to the SENSE program.

Data Collection Category	Variable	Phase 1 (Baseline Data)	Phase 2 (Time Interruption)	Phase 3 (Maintenance)
Delivery	Average number of times parent/guardian visits each day (range)	0.642 (0-3)	1.22 (0-3)	1.05 (0-3)
	Percentage of participants who have a caregiver visit each day (%)	55.77	82.22	66.65
	Average length of positive sensory input from parents/guardian, min (range)	43.88 (0-180)	98.67 (0-180)	92.7 (0-180)
	Percentage of bedsides with accurate SENSE materials hanging at bedside	95.6	100	100
	Number of times nurse provides positive sensory input	N/A	6	21
Education	Average number of times per week parents receive education from Occupational Therapist (range)	0.54 (0-2)	0.427 (0-1)	0.082 (0-1)
	Number of times SENSE is modeled by Occupational Therapist to staff	N/A	7.25	10.62
Adherence	Percentage of participants each week who received the full recommended amount of tactile sensory input (%)	10.16	46.75	43

Abbreviations: N/A, not applicable; SENSE, Supporting and Enhancing Neonatal Intensive Care Unit Sensory Experiences.

### Education on SENSE

All nurses who completed the preintervention survey stated that they had noticed the SENSE program signage. Eighty percent of nurses surveyed stated that the infants do not receive adequate positive sensory experiences. After education, 10 of 10 nurses who completed the postintervention survey were supportive of SENSE and staff participation (see Appendix 1 for the statements). The same 10 individuals did not complete the survey pre- and postintervention because of availability and work schedules. The occupational therapist used modeling an average of 7.25 times per week during phase 2 and increased to an average of 10.62 times per week during the final phase. The occupational therapist completed the most parent/guardian education during the first phase, with an average of 0.54 times per week (or once every other week) for each family. Table 4 shows the average data on these variables and the supplement for individual data.

### Adherence to SENSE

**Fig 2:**
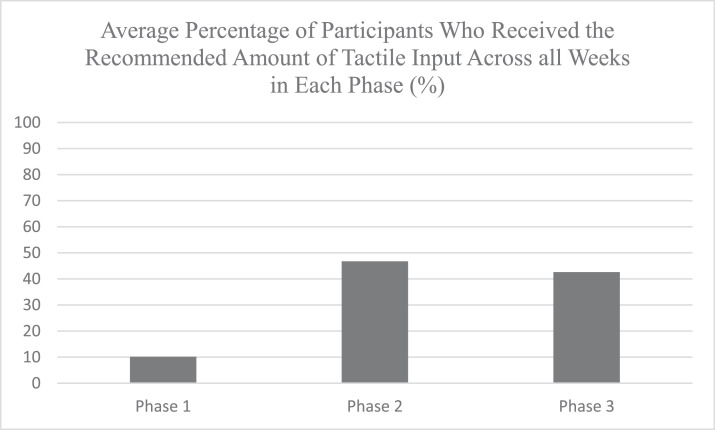
Average Percentage of Participants Who Received the Recommended Amount of Tactile Input Across all Weeks in Each Phase (%). Percentages of met tactile sensory recommendations were calculated for each week and then averaged across all weeks in each phase of the project.

Positive tactile input recommendations were met an average of 10% of the time during phase 1 (range, 0-35%), 50.75% of the time during phase 2 (range, 24%-67%), and 46.75% of the time during phase 3 (range, 10%-75%). Table 4 shows the average data of these variables and the supplement contains individual data, and figure 2 shows a graph of these data.

## Discussion

### Summary

This project used evidence-based practice to increase positive sensory experiences for medically fragile infants by improving implementation of SENSE in a NICU. Positive tactile input delivered to infants increased from phase 1 to phase 3. Nursing education and training was successful as evidenced by positive survey results and increased nurses’ use of positive sensory (tactile) interventions. The high participation and retention rates among caregivers support continued program use. These findings are similar to a previous study by Pineda and colleagues^21^ that found high rates of acceptability among parents as well as increased confidence by parents using SENSE. Of note is that in our quality improvement project, the rates of participation were high even without the use of logbooks to track the length and type of sensory input. Furthermore, the high participation and retention rates are supported by recent research that found increases in maternal confidence related to use of SENSE.^19^

With increases in parent/guardian presence and participation, as well as staff participation, there was an increase in positive tactile experiences delivered from phase 1 to phase 3. Although this could be due in part to the nurses’ education, another possible reason for the increase in positive tactile experiences in phase 3 is that these infants were, on average, 2 weeks older than those in phase 1. Parents/guardians and nurses may have been more comfortable or able to provide tactile experiences to older infants.

In relation to parent/guardian education, the findings noted a decrease in educational sessions from the first to the final phase. Possible explanations include a lack of parent/guardian presence during daytime weekday hours or decreasing need for ongoing education among infants who participated across multiple phases. The higher amount of education in phase 1 aligns with a previous study,^21^ which found that parent education provided earlier in the infant's hospitalization was linked to better parent participation in SENSE. It would be expected that parents who are new to the NICU require more education than parents who have been using SENSE for several weeks (phases 2 and 3 for infants whose hospitalization lasted multiple phases).

### Interpretation

Improving SENSE implementation through education, training, and modeling increased the amount of tactile sensory experiences infants received. This is similar to findings from Kolodko et al^26^ who reported that interventions used in the Behavior Change Wheel are more effective than other interventions to increase program implementation. Continued use of training, education, and modeling, coupled with other interventions listed in the Behavior Change Wheel, could support ongoing expansion of SENSE.^27^

### Study limitations

There were several limitations to this project. First, participants whose families were unable to visit during the occupational therapist's hours were unable to be recruited; therefore, the rates of infants reaching the full dosage of sensory input is likely higher in this project than what might occur across all infants. Future studies could improve recruitment strategies for evenings and weekends. Additionally, sensory input data from day nurses were only collected during the occupational therapist's weekday hours. Data collection from nurses’ use of SENSE across day and night shifts would increase accuracy. Because nurses were aware the project was taking place, there is a chance that they made stronger efforts to deliver positive sensory inputs knowing more about the project. Furthermore, data were collected mostly via observation, which could limit accuracy. Data were only collected on tactile input, so it is unknown if infants were receiving adequate sensory input for the remaining sensory systems. These limitations could be mitigated with improved methods for data collection, such as increased online documentation (in the electronic medical record) and more variables included.

A final limitation is a potential for bias because the occupational therapist intervened and collected data. This was managed by adhering to patient plans of care for therapy (3-5 times per week) to ensure all nurses and families/caregivers (when present) were equally exposed to SENSE via education and modeling. Future studies could incorporate an external researcher or data collector to eliminate this bias.

### Future directions

Future projects could involve expanding education, modeling, and training to involve others from the health care team, which may improve program adherence. Furthermore, the occupational therapist was only able to provide education when the parent/guardian was present during daytime hours. Expanding SENSE across the health care team may allow others to provide parent/guardian education. Another approach to explore is to have volunteers deliver sensory experiences instead of medical staff.^21^^,^^28^ This would be especially important for infants who have low parent presence specifically in rural communities where traveling to the hospital daily may be challenging.

## Conclusions

This project exemplifies how education, modeling, and training nurses around SENSE can increase positive sensory experiences. The outcomes suggest continued use of SENSE with the opportunity to expand education to additional health care providers or volunteers.

## Appendix Supplementary materials

Appendix 1 Pre- and postintervention surveys Image, application 1

Image, application 2

## Footnotes

Supplementary material associated with this article can be found, in the online version, at doi:10.1016/j.arrct.2024.100377.
