# Supplementary material for: Enhancing Sensory Experiences for Infants Born Preterm: A Quality Improvement Project
Source: Arch Rehabil Res Clin Transl. 2024 Oct 26;6(4):100377. doi: 10.1016/j.arrct.2024.100377 (PMC11733994; doi:10.1016/j.arrct.2024.100377)
Supplement: Supplementary file 1 — Appendix 1 Pre- and postintervention surveys [file mmc1.docx]

Appendix 1

Pre-Education Survey

1. What is your profession?
2. Have you noticed the SENSE program signage hanging at infants’ bedsides?
3. Do you think our patients receive enough positive sensory experiences (play, being held, being read to/talked to, etc.)?

Post-Education Survey

1. The program will support the role of the parent in the NICU by providing appropriate activities to do with their baby.

Agree Disagree

1. This program is helpful for educating parents on preterm infant development and how they can help their baby.

Agree Disagree

1. The program is easy to follow and information at the bedside is helpful for parents and staff in the NICU.

Agree Disagree

1. I am more aware of appropriate activities for my patients because of this program.

Agree Disagree

1. If/when I have time, I want to/plan to help engage my patients in the activities recommended by the program.

Agree Disagree

1. I support continuing to use and expand the program in our unit.

Agree Disagree
