# Supplementary material for: Enhancing Sensory Experiences for Infants Born Preterm: A Quality Improvement Project
Source: Arch Rehabil Res Clin Transl. 2024 Oct 26;6(4):100377. doi: 10.1016/j.arrct.2024.100377 (PMC11733994; doi:10.1016/j.arrct.2024.100377)
Supplement: Supplementary file 2 [file mmc2.docx]

Supplemental Data. Averages for each week of the project for data related to delivery of, education on, and adherence to SENSE with adaptations.

|  | **Delivery of SENSE Program with Adaptations** | | | | |
| --- | --- | --- | --- | --- | --- |
| **Week** | **Average Number of times Caregiver visits each Day (Range)** | **Percentage of Participants who have a caregiver visit each day**  **(%)** | **Percentage of Bedsides with Accurate SENSE Materials Hanging at Bedside** | **Average Length of Positive Sensory Input from Parents**  **(Minutes) (Range)** | **Number of Times Nurse Delivers Positive Sensory Input Outside of Typical Care** |
| 1 | 0.902 (0-2) | 80 | 100 | 95 (0-120) | 0 |
| 2 | 0.688 (0-1) | 68.75 | 64.8 | 63.75 (0-120) | 0 |
| 3 | 0.47 (0-1) | 46.67 | 100 | 56 (0-180) | 0 |
| 4 | 0.214 (0-1) | 20.75 | 100 | 17 (0-120) | 2 |
| 5 | 0.30 (0-1) | 30 | 100 | 30 (0-120) | 0 |
| 6 | 0.10 (0-1) | 10 | 100 | 3 (0-30) | 4 |
| 7 | 1.4 (0-3) | 90 | 100 | 25 (0-60) | 0 |
| 8 | 1.06 (1-2) | 100 | 100 | 61.30 (0-180) | 0 |
| **Average** | **0.642 (0-3)** | **55.77** | **95.6** | **43.88 (0-180)** | **0.75** |
| 9 | 1.12 (0-3) | 82 | 100 | 80.4 (0-180) | 0 |
| 10 | 1.16 (0-3) | 76 | 100 | 76.4 (0-180) | 0 |
| 11 | 1.53 (0-3) | 86 | 100 | 125 (0-180) | 0 |
| 12 | 1.08 (0-2) | 83 | 100 | 112.9 (0-180) | 0 |
| **Average** | **1.22** | **82.22** | **100** | **98.675** | **0** |
| 13 | 1.05 (0-3) | 84 | 100 | 109.4 (0-180) | 1 |
| 14 | 0.84 (0-2) | 60 | 100 | 68.8 (0-180) | 6 |
| 15 | 1.12 (0-3) | 68 | 100 | 105.6 (0-180) | 1 |
| 16 | 1.14 (0-2) | 72 | 100 | 117.9 (0-180) | 5 |
| 17 | 1.5 (0-4) | 75.4 | 100 | 126.82 (0-180) | 5 |
| 18 | 1.25 (0-3) | 50 | 100 | 90 (0-180) | 1 |
| 19 | 0.83 (0-3) | 50 | 100 | 40 (0-180) | 2 |
| 20 | 0.67 (0-1) | 66 | 100 | 83 (0-180) | 0 |
| **Average** | **1.05** | **66.65** | **100** | **92.7** | **2.625** |

| **Education on SENSE Program with Adaptations** | | |
| --- | --- | --- |
| **Week** | **Average Number of Times Per Week Parents Receive Education from Occupational Therapist (Range)** | **Number of Times SENSE with Adaptations is modelled by Occupational Therapist to Staff** |
| 1 | 1.5 (1-2) |  |
| 2 | 0.5 (0-1) |  |
| 3 | 0.2 (0-1) |  |
| 4 | 0 |  |
| 5 | 0.5 (0-1) |  |
| 6 | 0 |  |
| 7 | 1 (1-1) |  |
| 8 | 0.6 (0-2) |  |
| **Average** | **0.54 (0-2)** |  |
| 9 | 0.4 (0-1) | 10 |
| 10 | 0.326 (0-1) | 5 |
| 11 | 0.73 (0-1) | 11 |
| 12 | 0.25 (0-1) | 3 |
| **Average** | **0.427 (0-1)** | **7.25** |
| 13 | 0.083 (0-1) | 11 |
| 14 | 0.095 (0-1) | 14 |
| 15 | 0.12 (0-1) | 13 |
| 16 | 0.034 (0-1) | 17 |
| 17 | 0 | 13 |
| 18 | 0 | 2 |
| 19 | 0.25 (0-1) | 5 |
| 20 | 0.07 (0-1) | 10 |
| **Average** | **0.082 (0-1)** | **10.62** |

| **Adherence to SENSE Program with Adaptations** | |
| --- | --- |
| **Week** | **Percentage of Participants each week who had the Recommended Amount of Sensory Input Received**  **(%)** |
| 1 | 33 |
| 2 | 35 |
| 3 | 13.3 |
| 4 | 0 |
| 5 | 0 |
| 6 | 0 |
| 7 | 0 |
| 8 | 0 |
| **Average** | **10.16** |
| 9 | 24 |
| 10 | 46 |
| 11 | 67 |
| 12 | 50 |
| **Average** | **46.75** |
| 13 | 49 |
| 14 | 10 |
| 15 | 48 |
| 16 | 56 |
| 17 | 72 |
| 18 | 75 |
| 19 | 11 |
| 20 | 20 |
| **Average** | **43** |
